# Equilibrium states and stability of pre-tensioned adhesive tapes

**DOI:** 10.3762/bjnano.5.182

**Published:** 2014-10-07

**Authors:** Carmine Putignano, Luciano Afferrante, Luigi Mangialardi, Giuseppe Carbone

**Affiliations:** 1 Dipartimento di Meccanica, Matematica e Management (DMMM), Politecnico di Bari, V.le Japigia, 185, 70125, Bari, Italy, http://tribolab.poliba.it; 2 Department of Mechanical Engineering, Imperial College London, London SW7 2AY, United Kingdom

**Keywords:** adhesion, double-peeling, energy release rate, peeling of pre-tensioned tape

## Abstract

In the present paper we propose a generalization of the model developed in Afferrante, L.; Carbone, G.; Demelio, G.; Pugno, N. *Tribol. Lett.* **2013,** *52,* 439–447 to take into account the effect of the pre-tension in the tape. A detailed analysis of the peeling process shows the existence of two possible detachment regimes: one being stable and the other being unstable, depending on the initial configuration of the tape. In the stability region, as the peeling process advances, the peeling angle reaches a limiting value, which only depends on the geometry, on the elastic modulus of the tape and on the surface energy of adhesion. Vice versa, in the unstable region, depending on the initial conditions of the system, the tape can evolve towards a state of complete detachment or fail before reaching a state of equilibrium with complete adhesion. We find that the presence of pre-tension in the tape does not modify the stability behavior of the system, but significantly affects the pull-off force which can be sustained by the tape before complete detachment. Moreover, above a critical value of the pre-tension, which depends on the surface energy of adhesion, the tape will tend to spontaneously detach from the substrate. In this case, an external force is necessary to avoid spontaneous detachment and make the tape adhering to the substrate.

## Introduction

The understanding of adhesion of thin films is of prominent importance in a huge number of biological and biomechanical applications. As an example, the extraordinary adhesive abilities characterizing the hairy attachment systems of insects, reptiles and spiders have drawn significant research efforts aimed at reproducing such properties in artificial bio-mimetic adhesives [1–3]. In nature, many adhesive systems consist of arrays of hierarchical hairs or setae, enabling large contact areas and hence high adhesion owing to the van der Waals interaction forces [4]. This morphology enables many insects, spiders and some vertebrates to climb on almost any surface, from smooth ones to cinder block-like surfaces [5]. In this respect, it has been shown in [6–7] that the highly flexible terminal spatula elements, which behave as compliant contacting surfaces, play a crucial role in the adhesion. Many efforts have been taken trying to reproduce these structures to enhance adhesion and realize bio-inspired systems that could be employed, for example, in industrial material processing or as innovative smart solutions in structural design [8].

For these reasons, the mechanism of adhesion and detachment of systems such as thin films have been investigated by many experimental [9–13] and theoretical [3,14–24] approaches. However, many issues are not yet clear and our knowledge on this topic is still far from being complete. For example, in spite of several theoretical investigations about rough contact mechanics [25–29], the role of roughness in this kind of systems is not yet well understood. Furthermore, viscoelasticity, which entails prominent effects in terms of friction and contact anisotropy [30–31], has not yet been included in analytical and numerical models. In nature, on the other side, geckos exhibit extremely high adhesive performance also on rough substrates. The secret of this amazing behavior is mainly related to the fibrillar hierarchical geometry of the adhesion pads that makes these structures very compliant, despite the fact that they are usually constituted mainly of a relatively stiff material, namely β-keratin. The study of the mechanism of detachment of thin films can also help to elucidate some aspect of insects and, in particular, gecko adhesion. To avoid toe detachment, the gecko often employs the use of opposing feet and toes leading to a V-shaped geometry [9–10,32–35], which can be modelled by multiple-peeling schemes, as shown in [15,17,36], in which, based on the ground-breaking analysis proposed by Kendall [37], the crucial role of the spatula-shaped terminal elements in the biological hairy adhesive systems is pointed out.

In this paper, we focus our attention on some yet unclear aspects of the peeling process and, in particular, on the stability of this mechanism in presence of a pre-tension.

### The peeling process of a thin elastic tape

**Figure 1:**
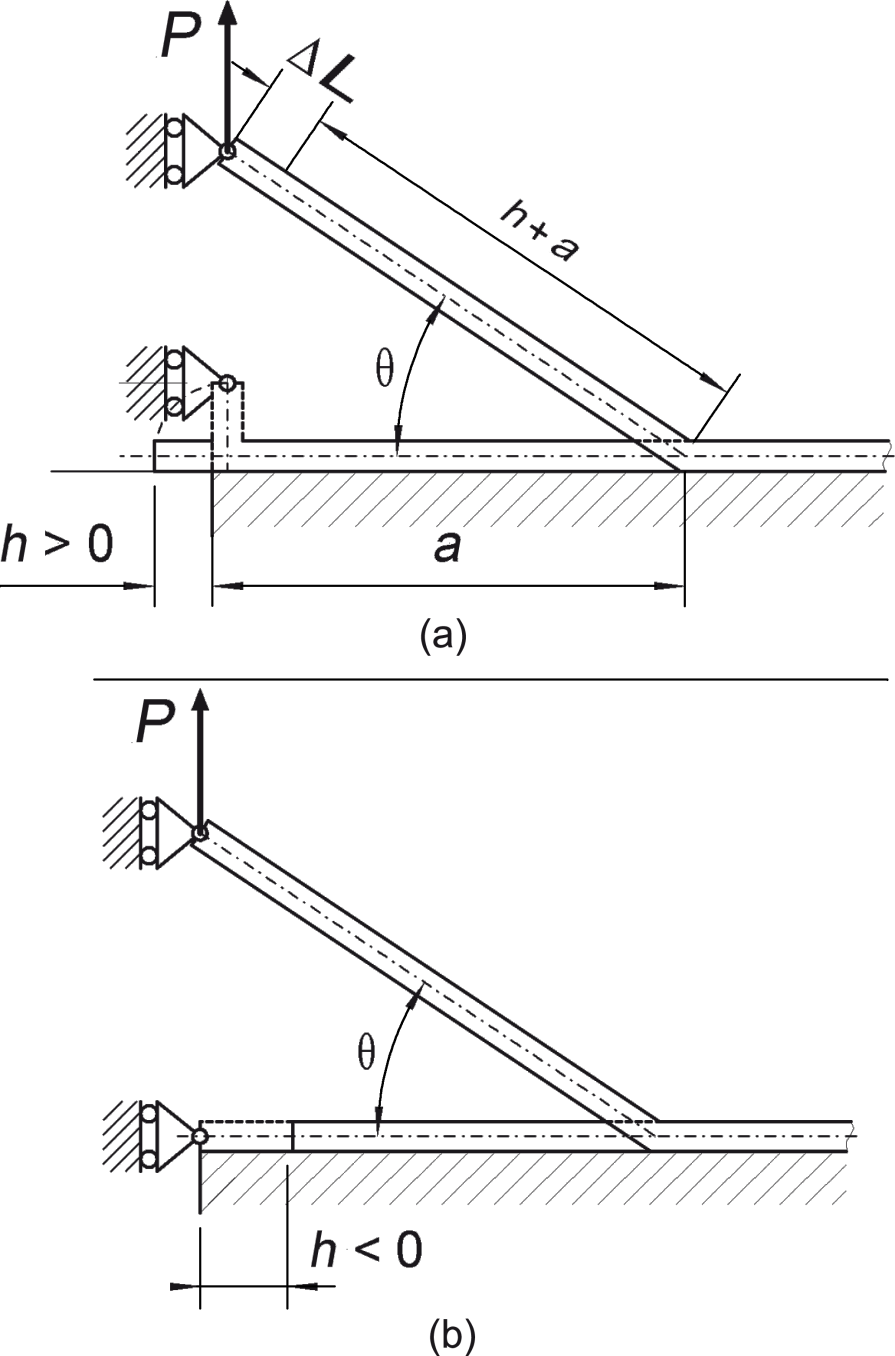
Double peeling of a tape. Initial configuration (a): a length *h* of the tape is not attached to the substrate and it is rotated before applying the external force *P*; initial configuration (b): the tape is stretched of a quantity *h* before loading.

In this section, we extend the formulation given in [36], focusing our attention on the stability analysis of the peeling process and on the effect of pre-tension on the mechanism of detachment. The formulation of the problem is developed considering two different initial configurations of an elastic tape with cross section *A* = *bt*, as shown in Figure 1. In the first configuration (Figure 1a) a portion of the tape length *h* is not attached to the substrate and it is rotated before applying the external force *P*. In the latter (Figure 1b) the tape has to be stretched by a quantity *h* before loading. In both cases, the tape can be pre-tensioned before being attached to the substrate. Incidentally, this loading procedure, with the force acting along the vertical axis and the edge of the tape being constrained to move along the same direction, is interesting because the double-peeling mechanism, due to symmetry, can be reduced to a scheme of this type [36].

During the peeling process the non-contact area is modeled as an interfacial crack, which determines the peeling advance as it propagates. The tape is assumed to be linearly elastic and incompressible.

A vertical force *P* is applied to the edge of the tape, as shown in Figure 1, and increases the length of the tape by a quantity

[1]
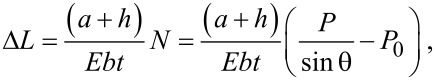


where *N* = *P*/sinθ − *P*_0_ is the normal force acting along the tape axis, *P*_0_ is the pre-tension and *E* is the Young modulus.

The change of the elastic energy stored in the system is

[2]
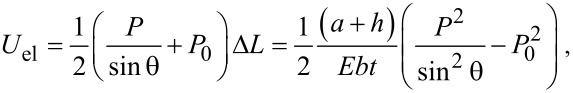


and the potential energy, which is the opposite of the work done by the external force *P*, is

[3]
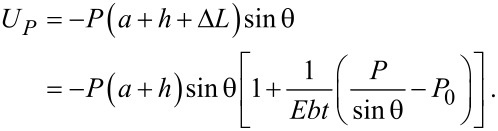


Equilibrium requires the stationarity of the total energy *U*_tot_, i.e., according to the Griffith criterion

[4]
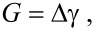


where Δγ is the Dupré energy of adhesion [38], and *G* is the energy release rate at the crack tip, defined as

[5]
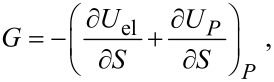


when the external load *P* is given. In Equation 5 *S* is the detached area.

Before solving the problem it is convenient to introduce the following dimensionless quantities

[6]
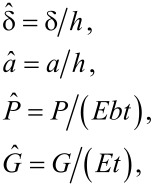


where we have defined δ through the relation δ + *h* = (*a* + *h* + Δ*L*) sinθ. From Equation 5 we obtain the following expression for the dimensionless energy release rate

[7]
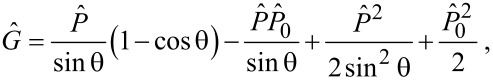


where we have used the geometric condition *a* = (*a* + *h* + Δ*L*) cosθ (see Figure 1), leading to

[8]
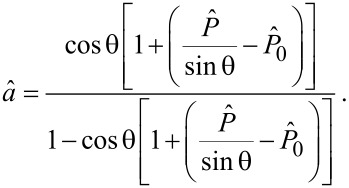


Note that Equation 7 is coherent with what was found in [39] for the single peeling of a pre-tensioned tape. Finally, from Equation 4 and Equation 7 the load 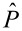 can be related to the peeling angle θ_eq_ at equilibrium and the corresponding dimensionless vertical displacement 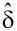 takes the form

[9]
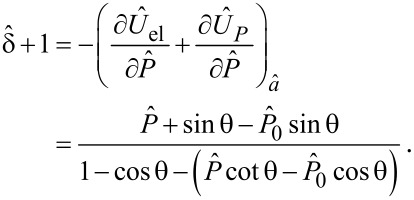


## Results and Discussion

In this section we discuss the influence of the pre-tension on the peeling process while paying particular attention to the detection of the critical transition thresholds between different regimes of detachment.

### Stability analysis of the peeling process

**Figure 2:**
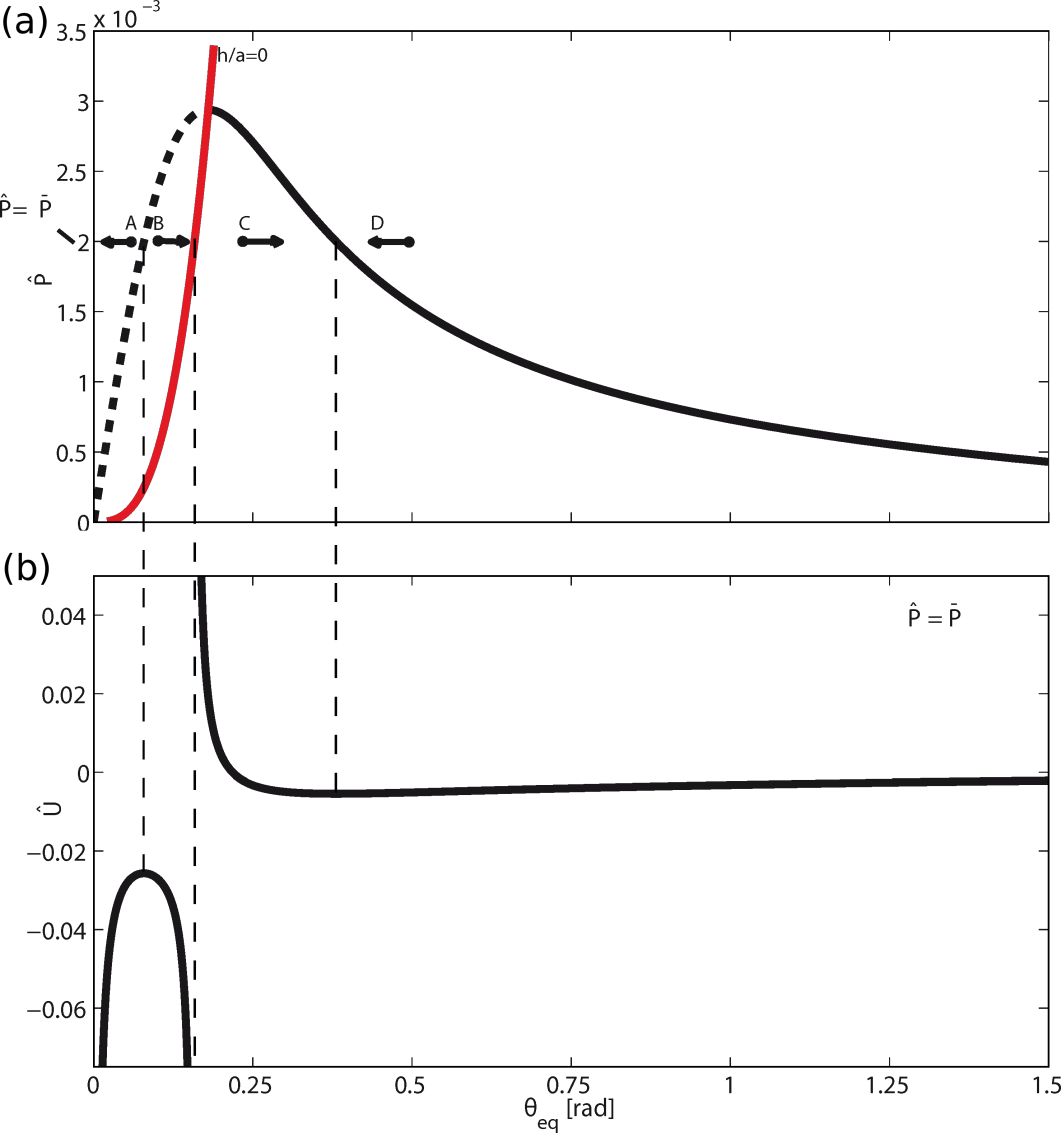
The dimensionless peeling force 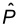 as a function of the peeling angle θ_eq_ at equilibrium (a); the total energy 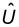 as a function of the peeling angle θ even out of equilibrium (b). For a fixed peeling force, depending on the initial configuration of the tape, the system can evolve towards states of partial adhesion (points *C* and *D*), complete detachment (point *B*) or can fail before reattaching to the substrate (point *A*).

Figure 2 shows the dimensionless peeling force 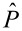 (Figure 2a) as a function of the peeling angle θ_eq_ at equilibrium, and the relative dimensionless total energy 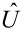 (Figure 2b) at a given load 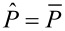 as a function of the peeling angle (even out of equilibrium). We observe that, for the considered case, given the applied load, two equilibrium states exist: one in the region *h*/*a* > 0 (corresponding to the tape configuration shown in Figure 1a), and the other in the region *h*/*a* < 0 (corresponding to the configuration in Figure 1b). In the latter case (*h*/*a* < 0), the equilibrium (dashed line in Figure 2a) is unstable since it corresponds to a maximum of the total energy 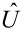 (see Figure 2b). Vice versa, in the region *h*/*a* > 0, the total energy 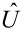 takes a local minimum at the peeling angles solving Equation 4 and, therefore, the corresponding configurations (solid line in Figure 2a) are stable.

In order to investigate what happens at a fixed pull-off force 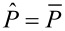, when the system is initially in non-equilibrium conditions, let us consider the starting configurations *A*, *B*, *C* and *D* shown in Figure 2a. Starting from point *A*, the tape evolves towards smaller and smaller peeling angles in order to minimize the total energy. At the end of this process, the peeling angle vanishes. Really, such a configuration cannot be reached because for the vertical load *P* to be balanced an infinite stress in the tape would be necessary. Therefore, the tape will fail before adhering to the substrate. On the contrary, when the system starts from point *B*, the tape peeling angle increases until the red curve is touched and, as a result, the complete detachment of the tape occurs.

When the system initially moves from a non-equilibrium configuration in the region on the right side (points *C* and *D*), it will be always able to reach a stable equilibrium with a finite detached area, corresponding to the local minimum of the total energy.

The above results lead to the conclusion that solutions corresponding to the dashed curve of Figure 2a are physically admissible only when the tape is initially stretched (see Figure 1b). However, they are unstable. In fact, depending on the starting conditions, a small perturbation can bring the tape to failure (point *A* of Figure 2a) or to complete detachment from the substrate (point *B* of Figure 2a). In particular, the condition *h*/*a* = 0 defines a boundary that separates stable and unstable regions. Notice these results are coherent with what was found in [36].

### Effect of pre-tension

**Figure 3:**
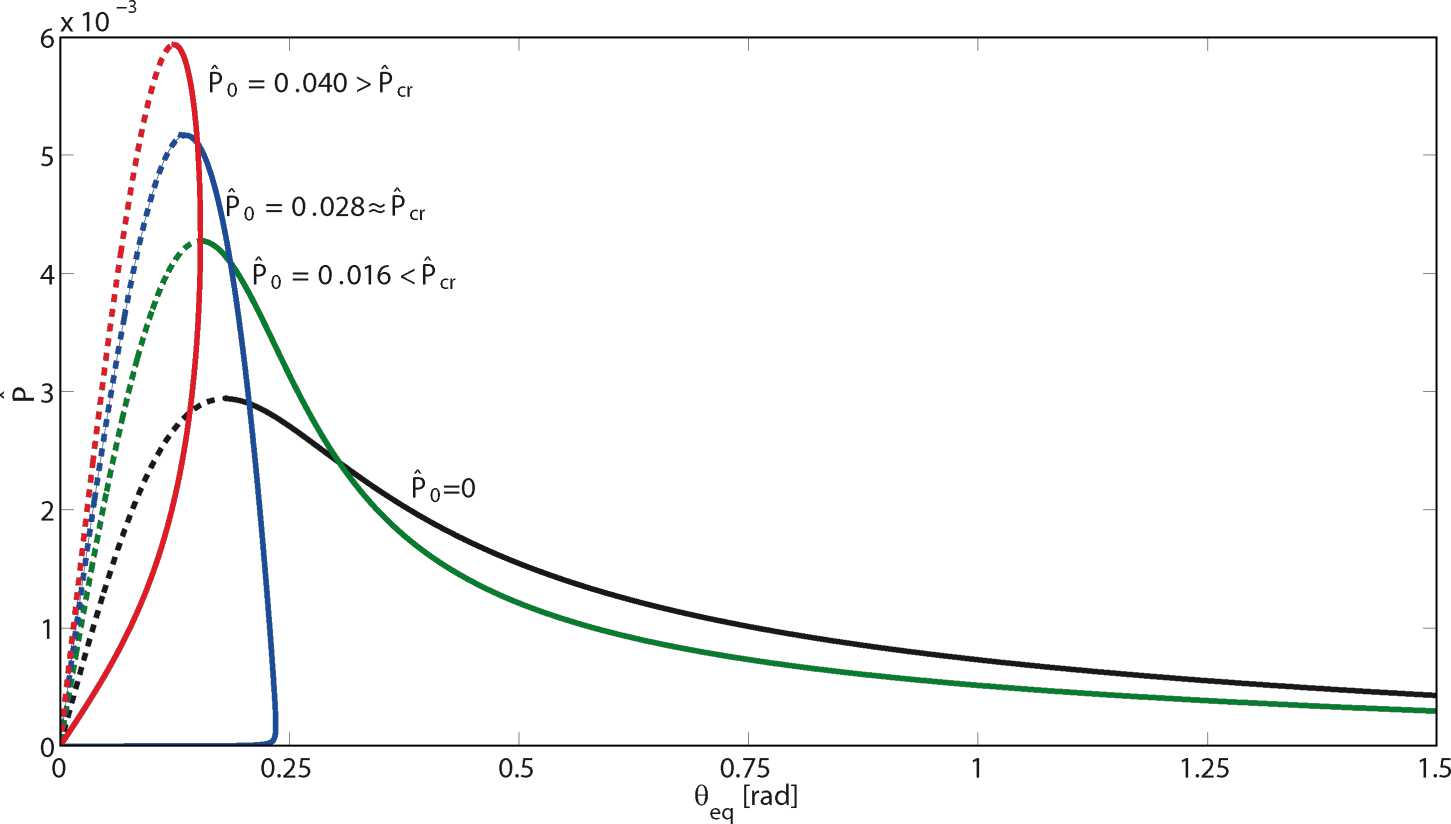
The dimensionless peeling force 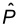 as a function of the peeling angle θ_eq_ at equilibrium, for different values of the dimensionless pre-tension 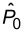. The pre-tension generally increases the pull-off force at low peeling angles.

First of all, we observe that the presence of a pre-tension *P*_0_ does not modify the conclusions of the stability analysis in the above section. The pre-tension *P*_0_ only affects the boundary *h*/*a* = 0 between stable and unstable regions. Figure 3 shows the dimensionless pull-off force 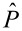 as a function of the peeling angle θ_eq_ at equilibrium, for different dimensionless values of 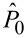. Again, unstable solutions are plotted with dashed lines, and the stable ones with solid lines. Note that the maximum pull-off force 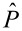 that can be sustained by the tape increases with the pre-tension 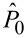, and correspondingly the lower bound θ_lim_ of the peeling angle, at which the pull-off force takes its maximum value, reduces. However, a critical value 
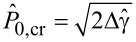
 of the pre-tension can be identified, above which the tape spontaneously detaches without applying any external vertical force. Indeed, when the pre-tension 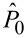 exceeds the threshold 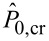, the tape spontaneously detaches. Interestingly, in this case, finite values of the pull-off force 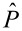 are necessary to make the system adhering to the surface. Furthermore, above 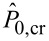 the peeling angle cannot exceed a critical value θ_cr_.

**Figure 4:**
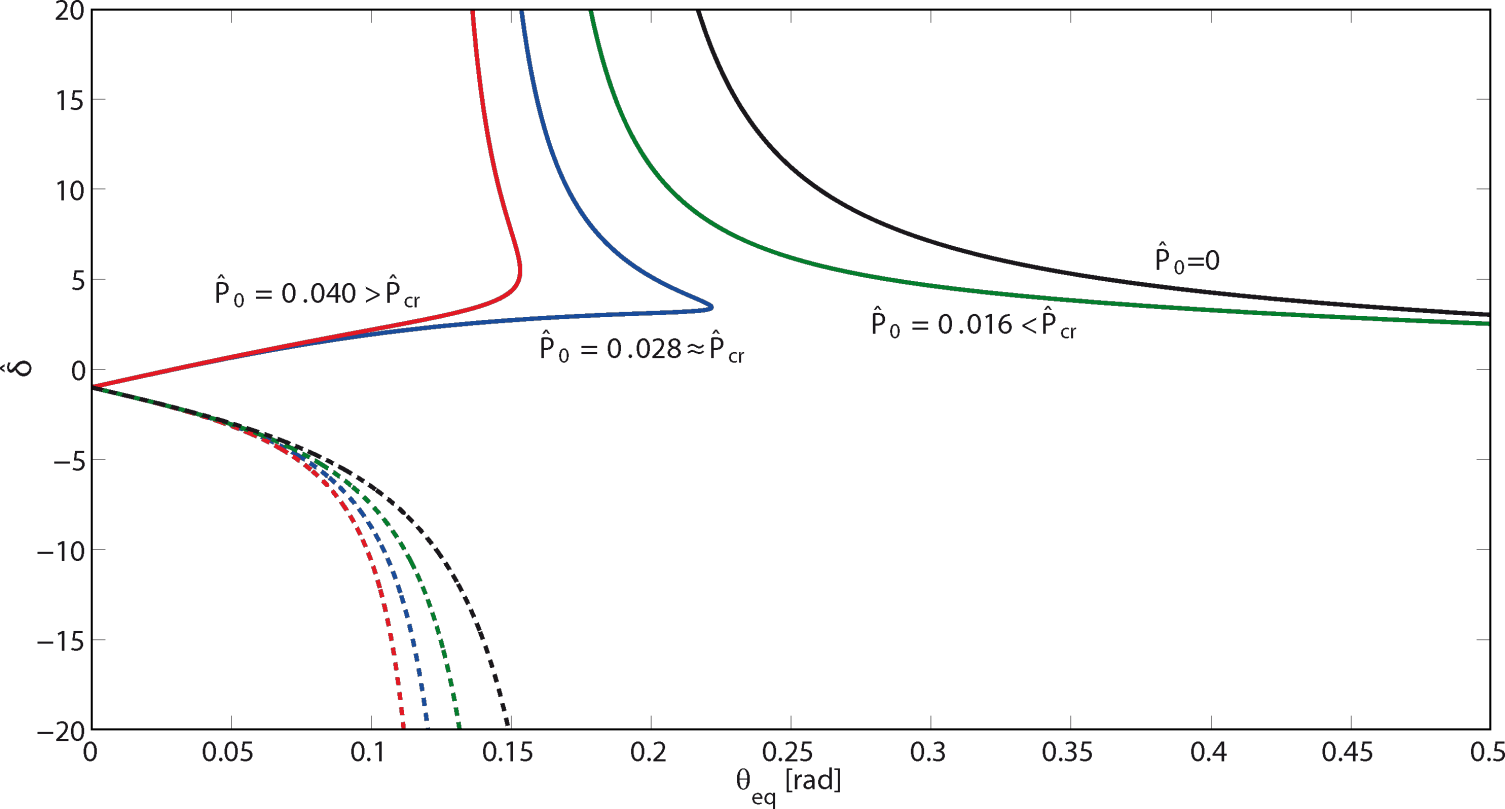
The dimensionless displacement 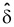 as a function of the peeling angle θ_eq_ at equilibrium, for different values of the dimensionless pre-tension 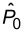.

Figure 4 shows the variation of the dimensionless displacement 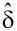 with the peeling angle θ_eq_ at equilibrium. Stable solutions are plotted with solid lines, the unstable ones with dashed lines. The displacement diverges as the peeling angle approaches θ_lim_, so at the maximum pull-off force the corresponding displacement is infinite, and this explains why with a finite force we can detach a tape of infinite length. Observe that at θ_eq_ = 0 the term δ + *h* necessarily vanishes and, thus, 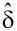 = −1. Moreover, Figure 4 shows that on the unstable branches (dashed lines) the dimensionless displacement 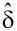 < −1. This means that on the unstable branches the quantity *h* is negative, and the configuration of the tape is the one represented in Figure 1b.

## Conclusion

The mechanism of detachment of an elastic thin tape adhering to a rigid substrate has been investigated, generalizing the model proposed in [36] with the incorporation of pre-tension in the tape and by performing an equilibrium stability analysis. Two equilibrium states are found: one being stable, the other being unstable. The two regimes strictly depend on the initial conditions of the system. In particular, solutions on the unstable branch are possible only when the tape is locally stretched before applying the pull-off force. In this case, if the starting point is at the left side of the unstable curve, in order to minimize the total energy, the peeling angle decreases until it vanishes. At the same time, in order to balance the applied vertical load, the stress in the tape increases and at zero peeling angle it should diverge. However, the tape, not being able to support infinite loads, necessarily fails before the new full adhesive equilibrium state can be reached. If the starting condition is at the right side of the unstable curve, the tape evolves towards a new state involving complete detachment.

Pre-tension does not change the above conclusions on the equilibrium stability. However, a pre-tensioned tape can sustain higher values of the pull-off force, before complete detachment. Interestingly, we find that above a critical value of the pre-tension, the tape cannot spontaneously adhere to the substrate, and an external load is therefore necessary to prevent spontaneous detachment.
